# DIVARICATA1 Promotes Leaf Degreening and Senescence in Arabidopsis

**DOI:** 10.3390/plants15081189

**Published:** 2026-04-13

**Authors:** Xumin Wang, Da Zhang, Chao Zhang, Jianchao Sun, Xinmei Ji, Zhuo Yan, Zhenqing Xia, Jianchao Cui, Shiyuan Liu, Chaohong Zhang, Yongjie Wu

**Affiliations:** 1 Changli Institute of Pomology, Hebei Academy of Agriculture and Forestry Sciences, Qinhuangdao 066600, China; 15383822971@163.com (X.W.); d.zhang@nwafu.edu.cn (D.Z.); 18838933981@163.com (C.Z.); jxm1008@163.com (X.J.); 13731396625@163.com (Z.Y.); 17854233612@163.com (Z.X.); cjc19880320@126.com (J.C.); liushiyuan123123@163.com (S.L.); 2 Bureau of Agriculture and Rural Affairs of Weichang Manchu and Mongolian Autonomous County, Chengde 068450, China; 19903249120@163.com

**Keywords:** DIV1, NAC, transcription factor, leaf senescence, GA

## Abstract

Leaf senescence is a critically regulated developmental process that determines crop yield and quality. MYB and NAC transcription factors (TFs) are central regulators within this network, yet the crosstalk between these TF families and their connection to the gibberellin (GA) pathway remain poorly understood. This study revealed that overexpression of *DIV1*, a MYB-like TF, leads to significantly reduced chlorophyll content and precocious leaf senescence. Based on the public transcriptome profiling of *DIV1*-overexpression leaves, 37 senescence-associated differentially expressed genes (DEGs), including the highly upregulated *NAC59* and *NAC92*, were identified. Molecular assays confirmed that DIV1 directly binds to the promoters of *NAC59* and *NAC92* and activates their transcription. Meanwhile, yeast two-hybrid and split-luciferase assays demonstrated that DIV1 physically interacts with the GA biosynthetic enzyme *ent*-kaurene oxidase (KO1) both in vitro and in vivo. The promoted senescence phenotype in DIV1-overexpression lines was rescued by treatment with paclobutrazol (PAC), a GA biosynthesis inhibitor. In summary, our findings reveal a dual mechanism whereby DIV1 integrates the GA pathway and NAC-mediated transcription to regulate leaf senescence. This work provides new insights into the coordination between MYB and NAC TFs during hormone-mediated senescence.

## 1. Introduction

Crop yield and quality are primary objectives in agricultural and horticultural production, both fundamentally linked to leaf functionality [1]. Leaf senescence, the terminal phase of leaf development, involves chlorophyll degradation, macromolecule breakdown, and nutrient remobilization [2]. This process directly governs photosynthetic capacity and the levels of key phytochemicals that determine postharvest quality [1,3]. While senescence follows an age-dependent program regulated by endogenous networks, it is also modulated by environmental and hormonal cues such as salinity, drought, high temperature, ethylene (ET), and gibberellic acid (GA) [4]. Therefore, investigating leaf senescence and developing strategies to modulate it will elucidate its underlying mechanisms and provide valuable insights for enhancing agricultural productivity.

MYB and NAC transcription factors (TFs) have been established as central regulators within the stress- and hormone-mediated network that controls chlorophyll degradation and leaf senescence [5]. For instance, MYB2 promotes whole-plant senescence by repressing cytokinin (CK) production [6]. Another R2R3-MYB TF, MYB44, interacts with the abscisic acid (ABA) receptor PYL8 to modulate ABA signaling, thereby regulating the expression of multiple senescence-associated genes (SAGs) and ultimately delaying leaf senescence [7]. MYB59 negatively regulates salicylic acid (SA)- and jasmonic acid (JA)-mediated leaf senescence by directly repressing the expression of *ICS1*, *PAL2*, and *LOX2* [8]. MYBH, a single-repeat MYB TF, accelerates leaf senescence by repressing auxin-amido synthase genes *GH3.6* and *GH3.10*, and upregulating *SAUR36*, a regulator of auxin-promoted leaf senescence [9]. Among NAC TFs, the ABA-responsive VNI2 couples ABA-mediated abiotic stress signals to leaf senescence by modulating a subset of *COR* and *RD* genes [10]. Rapidly induced by H_2_O_2_, *JUB1* overexpression is sufficient to delay leaf senescence and increase plant resilience to diverse abiotic stresses [11]. NTL4 promotes the expression of *RBOHD* genes, inducing ROS accumulation and triggering leaf senescence in response to drought stress [12]. Similarly, NAC32 promotes leaf senescence under abiotic stress by regulating *NYE1* and *SAG113*, which are involved in chlorophyll degradation and ABA signaling, respectively [13]. Furthermore, *NAC59* is rapidly and strongly induced by H_2_O_2_, and its overexpression accelerates senescence in transgenic plants [14]. NAC92, which co-evolves with NAC59, positively regulates ABA-, ET-, and JA-mediated leaf senescence [15], and its precise molecular mechanisms have since been extensively investigated. ATAF1 regulates senescence by both activating the ABA biosynthetic gene *NCED3* and repressing the chloroplast maintenance transcription factor *GLK1*, a function for which *NAC92* is required [16]. EIN3, the master positive modulator of ET signaling, directly promotes chlorophyll degradation by inducing the expression of *NYE1*, *NYC1*, *PAO*, and *NAC92*. Meanwhile, NAC92 also promotes the expression of a similar set of chlorophyll catabolic genes directly [17]. On the other hand, it has been demonstrated that removal of DELLA repression promotes leaf senescence and upregulates certain genes in various hormone pathways, suggesting that GA signaling acts upstream of the JA, SA, and ET pathways in regulating leaf senescence [18]. The studies above illustrate the pivotal roles of MYB and NAC TFs. However, key questions remain, particularly regarding the extent of crosstalk between the MYB and NAC families, and the role of MYB factors in GA-mediated senescence.

Previous studies discovered that DIVARICATA1 (DIV1), an R-R-type MYB [19], plays important roles in several biological processes during plant growth and development in *A. thaliana*. It promotes flowering through regulating GA biosynthesis and flowering integrators [20]. DIV1, directly targeted by NF-YC9, positively modulates seed germination in response to salinity stress by regulating the expression of *DOGL3* and *GASA4* [21]. Additionally, DIV1 negatively regulates *PIN5* expression to modulate primary root growth [22]. Moreover, analysis of publicly available gene expression data capturing the transition from non-photosynthetic to photosynthetic growth shows that *DIV1* was down-regulated during *A. thaliana* de-etiolation (greening) [23]. This inverse correlation with chlorophyll biogenesis prompts the hypothesis that DIV1 may play a positive role in the leaf degreening and senescence process.

In this study, it was found that DIV1 directly modulates the expression of *NAC59* and *NAC92* in developing leaves. Furthermore, DIV1 interacts with the GA biosynthetic enzyme KO1, and the degreening phenotype of DIV1-overexpression lines was rescued by paclobutrazol (PAC) treatment. Together, these results establish DIV1 as a key regulator that integrates the GA pathway and NAC-mediated transcription to control leaf degreening and senescence.

## 2. Results

### 2.1. DIV1 Drives Leaf Degreening and Triggers Global Repression of Chlorophyll Metabolism

**Figure 1:**
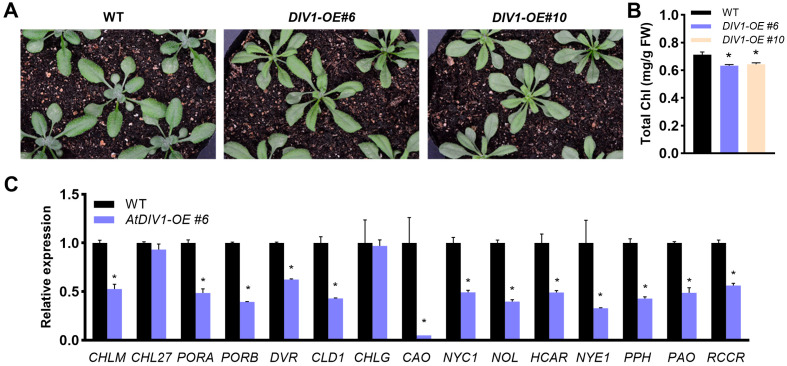
DIV1 promotes leaf degreening in *A. thaliana*. (**A**) Leaf degreening phenotype analysis between *DIV1-OE#6*, *#10* and wild type (WT) at 18 DAGs. OE, overexpression. (**B**) Statistical analysis of chlorophyll (Chl) content in the *DIV1-OE#6*, *#10* and wild type (WT) leaves at 18 DAGs. Values are means ± SD (n = 3). Asterisks indicate significant differences compared with the wild type (two-tailed paired Student’s *t*-test, *p* ≤ 0.05). Pairwise comparisons between the overexpression lines were not performed. (**C**) RT-qPCR analysis of the expression of chlorophyll metabolism genes in the *DIV1-OE#6* and wild type (WT) leaves at 8 DAGs. Results were normalized against the expression of *EF1αA4* as an internal control. Values are means ± SD (n = 3). Asterisks indicate significant differences compared with the wild type (two-tailed paired Student’s *t*-test, *p* ≤ 0.05).

*DIV1* was highly expressed in developing rosette leaves, as indicated by the ePlant database (https://bar.utoronto.ca/eplant/ (accessed on 13 February 2026)), and as further confirmed by RT-qPCR and GUS staining assays [20,21]. Thus, DIV1 was selected as a candidate for this study to elucidate its function in leaf degreening and senescence. To this end, the *div1-1* and *div1-2* loss-of-function mutants, along with the *DIV1-OE#6* and *#10* overexpression lines, were employed for phenotypic analysis. To ensure uniformity in seed germination and subsequent seedling establishment, seeds of all genotypes used for phenotypic analysis were harvested simultaneously and stored under identical conditions. Careful observation revealed that the leaf phenotypes of *div1-1* and *div1-2* showed no clear differences in comparison with the wild type (Supplementary Figure S1). In contrast, the *DIV1-OE#6* and *#10* overexpression lines exhibited pale green leaves with a grayish hue at the time point of 18 days after germination (DAGs) (Figure 1A). Based on the observed pale green phenotype, chlorophyll content was quantified. The *DIV1-OE#6* and *#10* lines exhibited significantly lower chlorophyll levels compared to the wild type, and *DIV1-OE#6* was chosen for subsequent investigation (Figure 1B). Subsequently, the expression levels of key chlorophyll biosynthesis and catabolism genes were analyzed by RT-qPCR at 8 DAGs. The expression of chlorophyll biosynthesis genes (*CHLM*, *PORA*, *PORB*, *DVR*, *CLD1*, *CAO*) was significantly downregulated in the *DIV1-OE#6* line in comparison with the wild type, while *CHLG* and *CHL27* remained unchanged. Notably, contrary to expectations, key chlorophyll degradation genes (*NYC1*, *NOL*, *HCAR*, *NYE1*, *PPH*, *PAO*, *RCCR*) were also markedly downregulated (Figure 1C). This pattern further implies that the transcriptional regulation of these chlorophyll metabolic genes may not be directly mediated by DIV1.

### 2.2. DIV1 Promotes Leaf Senescence and Modulates a Series of Senescence-Associated Genes

**Figure 2:**
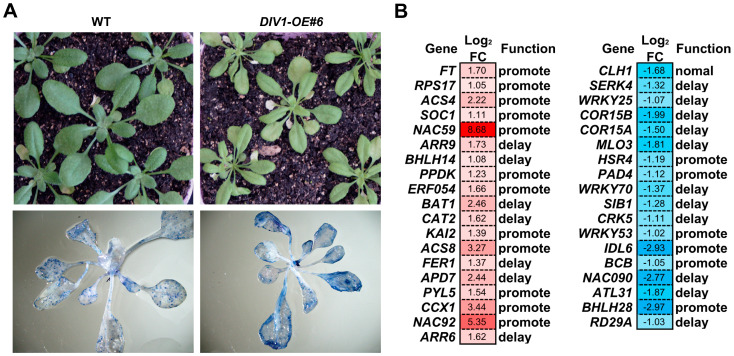
DIV1 promotes leaf senescence in *A. thaliana.* (**A**) Leaf senescence phenotype analysis between *DIV1-OE#6* and wild type (WT) at 22 DAGs. **Top panel**: plant photographs; **bottom panel**: trypan blue staining, scale bar = 200 μm. (**B**) Integrative analysis of transcriptome data with the leaf senescence database. Public transcriptome data from DIV1-overexpressing leaves [20] were compared against the leaf senescence database (https://ngdc.cncb.ac.cn/lsd/ (accessed on 13 February 2026)).

To determine whether DIV1 influences leaf senescence, further observations on the *DIV1-OE#6* plants were conducted. At 22 DAGs, *DIV1-OE#6* exhibited precocious senescence symptoms in rosette leaves compared to the wild type (Figure 2A, top panel). Trypan blue staining assay showed that cell death was more pronounced in the rosette leaves of *DIV1-OE#6* than in those of wild type plants (Figure 2A, bottom panel). To explore the molecular basis of this accelerated senescence, public transcriptome data from *DIV1*-overexpressing leaves [20] were analyzed against a leaf senescence reference database (https://ngdc.cncb.ac.cn/lsd/ (accessed on 13 February 2026)). This analysis identified 37 senescence-associated differentially expressed genes (DEGs), comprising 18 downregulated and 19 upregulated (Figure 2B; Supplementary Table S1). The expression trends of most DEGs (22/37) aligned with the observed *DIV1*-overexpression phenotype, further corroborating its senescence-promoting function. These included pathway-specific genes like *SOC1*, *PYL5*, and *WRKY70* (Figure 2B). To validate this finding, this study focused on the top two up-regulated genes, *NAC59* (log_2_FC = 8.68) and *NAC92* (log_2_FC = 5.35), and analyzed their expression via RT-qPCR in *DIV1-OE#6* and the wild type plants at 8 DAGs. The expression of *NAC59* and *NAC92* was upregulated in the leaves of *DIV1-OE#6* in comparison with the wild type, which were highly consistent with the transcriptome results (Supplementary Figure S2). In brief, the transcriptome and RT-qPCR results revealed that DIV1 may upregulate the expression of *NAC59* and *NAC92* to modulate leaf degreening and senescence.

### 2.3. DIV1 Directly Modulates the Expression of NAC59 and NAC92 in Developing Leaves

**Figure 3:**
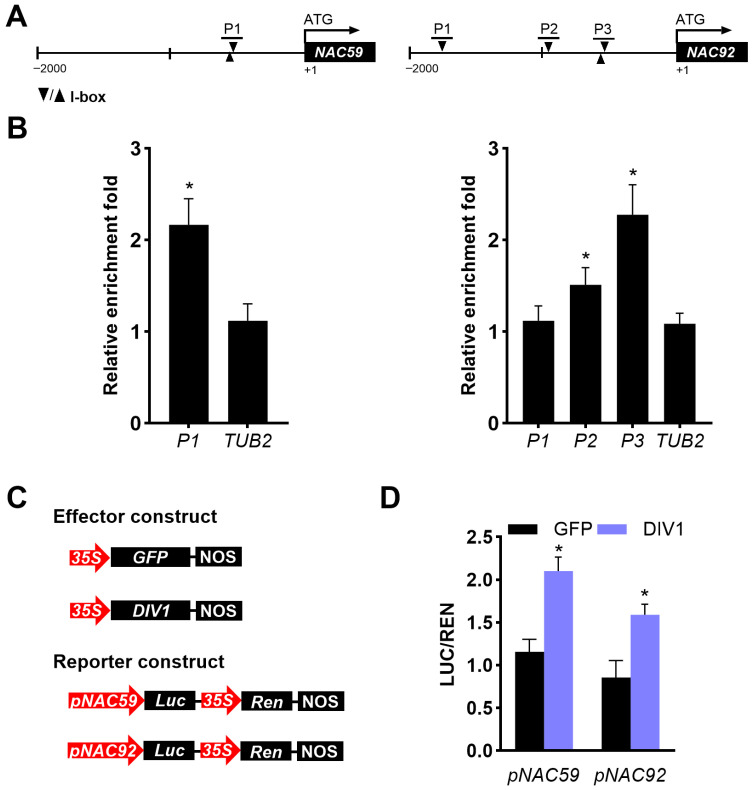
DIV1 targets *NAC59* and *NAC92* promoters and promotes their transcriptional activities. (**A**) Schematic representation of the *NAC59* and *NAC92* promoters. Exons are depicted as black boxes, while promoter regions are shown as black lines. PCR-amplified fragments are denoted by black lines positioned above the I-box motifs. (**B**) ChIP-qPCR analysis of DIV1 binding to the *NAC59* and *NAC92* promoters in developing leaves. Fold enrichment was calculated by normalizing target DNA amounts to an internal control (*EF1αA4*) and subsequently to the wild type level. A *TUB2* fragment was included as a negative control. Data are shown as mean ± SD (n = 3). Asterisks indicate significant differences compared with the *TUB2* fragment (two-tailed paired Student’s *t*-test, *p* ≤ 0.05). (**C**) Schematic diagrams show the effectors with GFP and DIV1 and the reporters containing *NAC59* and *NAC92* promoters. (**D**) Dual-luciferase assay showing DIV1-mediated activation of *NAC59* and *NAC92* promoters. Reporter and effector constructs were transiently co-expressed in *N. benthamiana* leaves. LUC activity was normalized to REN internal control, and fold induction was calculated relative to the GFP effector. Values represent mean ± SD (n = 9). Asterisks denote significant differences from the GFP control (two-tailed paired Student’s *t*-test, *p* ≤ 0.05).

DIV1 binds to the I-box motifs in *A. thaliana* [21]. Sequence analysis showed that the promoters of *NAC59* and *NAC92* contained I-box motifs (Figure 3A). To investigate how DIV1 regulates the expression of *NAC59* and *NAC92*, ChIP-qPCR assays were performed using the *DIV1-OE#6* leaves at 8 DAGs. The ChIP-qPCR results showed that DIV1 was associated with the promoter regions near fragments P1 of *NAC59*, and fragments P2 and P3 of *NAC92* (Figure 3B). Additionally, this work evaluated the transcriptional regulation of DIV1 on *NAC59* and *NAC92* through dual-luciferase reporter assay in *N. benthamiana* leaves. Constructs containing *NAC59* and *NAC92* promoter-driven LUC, and 35S promoter-driven REN were used as reporters. The pGreenII 62–SK recombinant vectors containing the CDSs of *GFP* and *DIV1* were used as effectors (Figure 3C). The DIV1 effector generated an increase in the *ProNAC59:LUC* and *ProNAC92:LUC* expression in comparison with the GFP control (Figure 3D). In short, these results collectively reveal that DIV1 binds to the promoters of *NAC59* and *NAC92*, thus directly regulating their expression to promote leaf degreening and senescence.

### 2.4. DIV1 Physically Interacts with KO1

**Figure 4:**
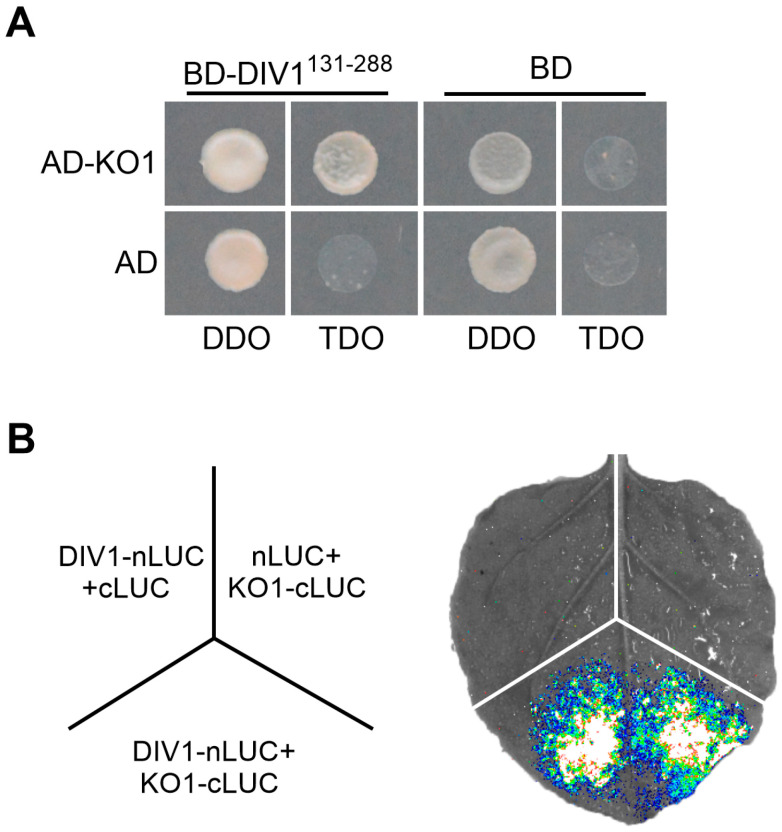
DIV1 interacts with KO1. (**A**) Y2H assay showing that DIV1 interacts with KO1 in yeast cells. DDO, D-Trp-Leu; TDO, SD-Trp-Leu-His. (**B**) Split-luciferase assay showing that DIV1 interacts with KO1 in tobacco leaves. Empty nLUC and cLUC constructs were used as negative controls.

To elucidate the in-depth mechanism underlying the *DIV1*-mediated regulation of leaf degreening and senescence, this work conducted yeast-two-hybrid (Y2H) screening to search for its putative interactors. Interestingly, the gibberellin biosynthetic enzyme KO1 was identified. First, the Y2H assay was used to verify the interaction between DIV1 and KO1. Co-expression of the DIV1-fused GAL4 DNA-binding domain (BD) and KO1-fused activation domain (AD) activated the *HIS3* reporter gene, indicating that DIV1 and KO1 physically interact in yeast (Figure 4A). Then, the split-luciferase assay showed that strong luciferase signals were detected only when DIV1 and KO1 were co-expressed in *N. benthamiana* leaves, further confirming the interaction between DIV1 and KO1 (Figure 4B). Collectively, these data imply that DIV1 interacts with KO1 both in vitro and in vivo.

### 2.5. DIV1-Mediated Leaf Degreening and Senescence Is Negatively Modulated by PAC

**Figure 5:**
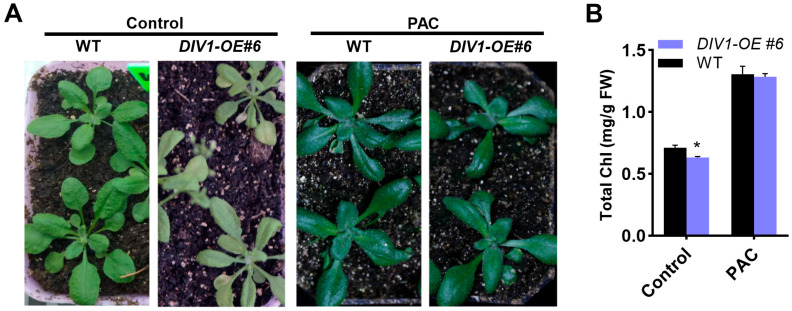
DIV1 positively regulates GA-mediated leaf degreening and senescence. (**A**) Leaf degreening and senescence phenotype of *DIV1-OE#6* and wild type (WT) at 22 DAGs under control and PAC treatment. (**B**) Statistical analysis of chlorophyll (Chl) content in the *DIV1-OE#6* and wild type (WT) leaves under control and PAC treatment. Values are means ± SD (n = 3). Asterisks indicate significant differences compared with the wild type (two-tailed paired Student’s *t*-test, *p* ≤ 0.05). PAC, Paclobutrazol.

Building on the protein interaction findings, the role of DIV1 in GA-mediated leaf degreening and senescence was further investigated. The *DIV1-OE#6* and wild type plants were treated with PAC, a KO1 inhibitor [24]. As shown in Figure 5, exogenous PAC application rescued the promoted leaf degreening and senescence phenotype of *DIV1-OE#6* to the wild type level. Therefore, the DIV1-mediated leaf degreening and senescence can be suppressed by inhibiting GA biosynthesis with PAC.

## 3. Discussion

The core objectives of agricultural and horticultural practices are to generate valuable plant-based biomass—including cereals, vegetables, fruits, and forages—with optimal yield and quality [25]. In most higher plants, leaves serve as the primary site of photosynthesis, a process whose efficiency and duration, alongside traits like inflorescence development and resource partitioning, are fundamental determinants of final crop productivity [26]. Although *DIV1* is highly expressed in developing leaves, particularly before the transition to reproductive growth, and has been established as a key modulator of flowering and seed germination [20,21], its function in leaf development remains unclear.

Leaf senescence is visually marked by a progressive loss of green pigmentation, reflecting the systematic breakdown of chlorophyll and the dismantling of chloroplast structures [27]. In this study, overexpression of *DIV1* promotes leaf degreening and senescence (Figure 1A and Figure 2A). However, neither the T-DNA insertion mutant (*div1-1*) nor the CRISPR/Cas9-generated mutant (*div1-2*) exhibited any visible leaf phenotype (Supplementary Figure S1), which may weaken the central claim that DIV1 is a key regulator of leaf senescence. In contrast, MYBS1 and MYBS2, two recently identified R-R-type MYBs belonging to the same subfamily as DIV1, were shown to positively regulate chlorophyll accumulation [23]. MYBS1 and MYBS2 positively regulate the expression of chlorophyll biosynthetic, light-harvesting and Calvin–Benson–Bassham cycle genes [23]. In terms of their roles in regulating leaf degreening and their downstream regulatory networks, MYBS1/MYBS2 differ significantly from DIV1, indicating functional divergence within this subfamily. Moreover, the *mybs1 mybs2* double mutant displayed pale leaves compared to the wild type, whereas the single mutants showed no obvious phenotypic alterations [23]. Similarly, previous studies have demonstrated that both DIV1 and DIV2 positively regulate seed germination in response to salinity stress [21,28], suggesting that DIVs may act redundantly in the control of leaf degreening and senescence. Further investigation through the generation and analysis of multiple mutants will be required to test this possibility.

Leaf senescence is an intricate process that necessitates the orchestrated expression of genes involved in aging, hormonal regulation, nutrient remobilization, and various other essential pathways [1,29]. The chlorophyll catabolic enzymes (CCEs) play important roles in this process [30]. During senescence, chlorophyll b is converted to chlorophyll a through the sequential action of chlorophyll b reductase (encoded by *NYC1*/*NOL*) and 7-hydroxymethyl chlorophyll a reductase (HCAR) [31,32,33,34]. The onset of chlorophyll a degradation is marked by magnesium dechelation and catalyzed by NYEs/SGRs, which yields pheophytin a [35]. This intermediate is subsequently metabolized via three enzymatic steps: pheophytin a is first dephytylated by pheophytin pheophorbide hydrolase (PPH) to generate pheophorbide *a* [36,37]; pheophorbide a is then oxygenated by pheophorbide a oxygenase (PAO) to form red chlorophyll catabolites (RCC); finally, RCC is reduced by red chlorophyll catabolite reductase (RCCR) to primary fluorescent chlorophyll catabolites [38]. The ring-opening step catalyzed by PAO, converting pheophorbide a to RCC, coincides with the loss of green color in chlorophyll catabolites [38]. Moreover, *NYE1* mutation is responsible for Mendel’s green cotyledon trait [39]. Notably, however, *DIV1* overexpression repressed the expression of *CCEs*, a response opposite to its degreening-promoting phenotype (Figure 1). This apparent contradiction may reflect the concurrent upregulation of some senescence suppressors and downregulation of senescence promoters in *DIV1*-overexpressing lines (Figure 2B). We speculate that these modulators might be responsible for the observed regulation of these genes. Meanwhile, the repression of chlorophyll biosynthetic genes in *DIV1-OE#6* provides a more straightforward explanation for the observed degreening phenotype (Figure 1C).

The transcriptional regulation of *NYE1* during degreening and senescence has been extensively studied. MYC2/3/4, EIN3, ABF2/3/4, PIF4, and NAC019/055/072 and NAC92 positively regulate the expression of *NYE1* and/or its paralog *NYE2* during leaf senescence in a hormone- and dark condition-dependent manner, whereas SOC1 negatively regulates the expression of *NYE1* [17,40,41,42,43,44,45]. It has been shown that DIV1 shares an expression pattern similar to that of NAC59 and NAC92, all of which are highly expressed in rosette leaves [14,20,46]. This finding is consistent with our results revealing that DIV1 directly and positively regulates the expression of *NAC59* and *NAC92* in developing leaves (Figure 3). Nevertheless, loss-of-function analyses of these NACs in the *DIV1-OE* background will be required in future studies to definitively establish their functional role in DIV1-mediated senescence. DIV1 also directly regulates the expression of *SOC1* in flowering control [20]. These findings suggest that DIV1 may act as an upstream regulator integrating multiple senescence-associated TFs to fine-tune leaf degreening and senescence, which could also account for the unexpected expression pattern of *CCEs* in the *DIV1*-overexpressing line.

DIV1 is involved in GA-mediated modulation of seed germination and flowering by influencing GA signaling and biosynthesis [20,21]. It has been demonstrated that GA biosynthetic enzymes are linked to leaf degreening and senescence [18]. In this study, we found that DIV1 interacts with GA biosynthetic enzyme KO1 (Figure 4), and PAC treatment rescued the degreening and senescence phenotype of *DIV1-OE#6* to the wild type level (Figure 5). The physical interaction between DIV1 (nuclear-localized [21]) and KO1 (plastid-localized [47]) presents an intriguing spatial paradox. We speculate that their interaction may be transient, condition-dependent, or involve protein shuttling between subcellular compartments. Whether this interaction affects KO1 enzymatic activity, stability, or subcellular dynamics remains to be determined. Future studies employing BiFC, enzyme activity assays, and protein stability analyses will be essential to elucidate the mechanistic details of this novel regulatory module. Collectively, our results position DIV1 as a previously unrecognized molecular linker that integrates GA pathway activity into the transcriptional network governing leaf degreening and senescence.

## 4. Materials and Methods

### 4.1. Plant Materials and Growth Conditions

The T-DNA insertion mutant *div1-1* (SALK_084867), the CRISPR/Cas9-induced mutant *div1-2* and *DIV1*-overexpression lines in the Col-0 background have been described previously [20]. All plants were grown at the same time in the same chamber under long-day conditions (16 h of light/8 h of dark) at 22 °C. The overhead light intensity was 160 μmol m^−2^ s^−1^, as detected at the middle region of the plants. For paclobutrazol (PAC) treatment, 7-day-old seedlings were sprayed twice per week with 25 μM PAC (a concentration sufficient to suppress GA-related processes such as germination and flowering, as determined in previous studies [20,48]) until the onset of bolting. PAC was first dissolved in a small volume of ethanol and then diluted with water to the working concentration.

### 4.2. Vector Construction

The CDS fragments of *DIV1* and *KO1* were amplified and separately cloned into pGBKT7 and pGADT7 (Clontech, Mountain View, CA, USA). The promoters of *NAC59* and *NAC92* were separately cloned into pGreenII 0800–LUC [49]. All constructs were generated using a high-fidelity enzyme KOD-Plus (TOYOBO, Osaka, Japan), and eight independent colonies per construct were sequenced by Sangon Biotechnology (Shanghai, China). Primers used for vector construction are listed in Supplementary Table S2.

### 4.3. Chlorophyll Content Measurement

Seedlings were weighed and immersed in 80% acetone in the dark at 4 °C for 24 h. The absorbance of the supernatant was measured at 663 nm and 645 nm using a spectrophotometer. Total chlorophyll contents were calculated as previously described [17].

### 4.4. Trypan Blue Staining

Whole seedlings were immersed in trypan blue staining solution, subjected to boiling water bath for 2 min, and then cooled to room temperature. Stained samples were destained in chloral hydrate solution for 2–3 days to remove background staining. Destained leaves were equilibrated with 70% glycerol and photographed under a stereomicroscope. Dead cells were visualized as blue spots.

### 4.5. Gene Expression Analysis

The total RNA was isolated with the Plant RNA Kit (TransGen, Beijing, China) and reverse transcribed using EasyScript One-Step gDNA Removal and cDNA Synthesis SuperMix (TransGen). RT-qPCR was conducted with three biological replicates using SYBR Green Mix (TransGen). Relative transcript levels were normalized to the internal reference gene *EF1αA4*. The expression levels of the selected genes were calculated using the 2^−ΔΔCt^ method. Primers used for gene expression analysis are listed in Supplementary Table S2.

### 4.6. ChIP-qPCR Assay

The ChIP assay was conducted as previously described [50]. Rosette leaves (5 g) from WT and *DIV1-OE#6* plants were harvested and cross-linked under vacuum for 15 min on ice in 1% formaldehyde, followed by quenching with 0.125 M glycine for 5 min. After two washes with distilled water, leaves were flash-frozen in liquid nitrogen. Chromatin was extracted and sonicated to generate DNA fragments of 200–700 bp. The sonicated chromatin was immunoprecipitated overnight at 4 °C with Pierce anti-HA magnetic beads (Thermo Fisher). Beads were collected and washed using a magnetic rack. Following two rounds of elution, the immune complexes were reverse-cross-linked at 65 °C for 10 h in 5 M NaCl. Proteins were digested with 0.5 M EDTA, 1 M Tris-HCl (pH 6.5), and 3 μL proteinase K (10 mg/mL) for 1 h at 45 °C. DNA was recovered by phenol/chloroform/isoamyl alcohol (25:24:1) extraction. Relative enrichment of each fragment was determined by RT-qPCR. Primers used for ChIP-qPCR are listed in Supplementary Table S2.

### 4.7. Dual-Luciferase Assay

Dual-luciferase assays were conducted following previously reported methods [50]. The DIV1 and GFP effector constructs, along with the *NAC59* and *NAC92* promoter-driven reporter constructs, were transformed into GV3101 (Weidi Biotechnology, Shanghai, China). Infiltrated *N. benthamiana* plants were cultured in a chamber under long-day conditions for 3 days prior to sampling. Leaf tissues were homogenized in Cell Lysis Buffer (YEASEN, Shanghai, China), and luciferase activities were measured using the Luciferase Reporter Gene Assay Kit (YEASEN) with an enzyme label instrument (Tecan, Männedorf, Switzerland).

### 4.8. Protein Interaction Assays

For Y2H assay, DIV1-BD and KO1-AD were co-transformed into Y2H Gold (Clontech) and grown on selection medium supplemented with SD/−Leu/−Trp and SD/−Leu/−Trp/−His to determine the interaction between DIV1 and KO1.

For the split-luciferase assay, the recombinant N-terminus/C-terminus of luciferase vector were transformed into GV3101 (Weidi Biotechnology) and co-injected into *N. benthamiana* leaves. Infiltrated plants were cultured in a chamber under long-day conditions for 3 days prior to sampling. Signals were captured using a low-light cooled CCD imaging system (Tanon, Shanghai, China).

## Data Availability

Accession numbers: Sequence data from this article can be found in the Arabidopsis Genome Initiative database under the following accession numbers: DIV1 (AT5G58900), NAC59 (AT3G29035), NAC92 (AT5G39610), KO1 (AT5G25900), CHLM (AT4G25080), PORA (AT5G54190), PORB (AT4G27440), DVR (AT5G18660), CLD1 (AT5G38520), CHLG (AT3G51820), CAO (AT2G47450), NYC1 (AT4G13250), NOL (AT5G04900), HCAR (AT1G04620), NYE1 (AT4G22920), PPH (AT5G13800), RCCR (AT4G37000).

## Supplementary Materials

The following supporting information can be downloaded at: https://www.mdpi.com/article/10.3390/plants15081189/s1. Figure S1. Loss of *DIV1* function does not alter leaf phenotype. Figure S2. RT-qPCR analysis of the expression of *NAC59* and *NAC92* in the *DIV1-OE#6* and wild type (WT) leaves. Table S1. Overlap between public *DIV1-OE* leaf transcriptome and senescence database genes. Table S2. Primers used in this study.
