# Supplementary material for: DIVARICATA1 Promotes Leaf Degreening and Senescence in Arabidopsis
Source: Plants (Basel). 2026 Apr 13;15(8):1189. doi: 10.3390/plants15081189 (PMC13119682; doi:10.3390/plants15081189)
Supplement: Supplementary file 1 [file plants-15-01189-s001.zip › Supplementary figures.pdf]

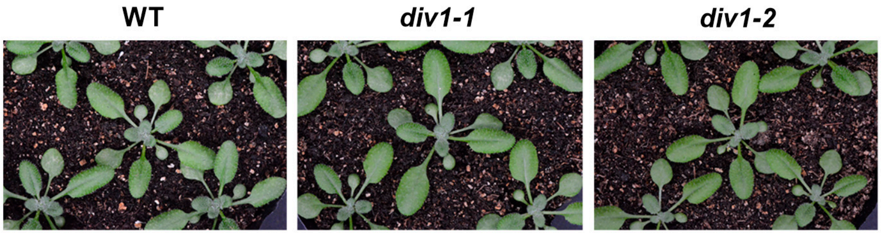

Supplemental Figure S1. Loss of *DIV1* function does not alter leaf phenotype.

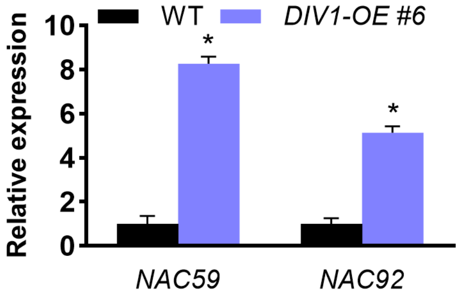

Supplemental Figure S2. RT-qPCR analysis of the expression of *NAC59* and *NAC92* in the *DIV1-OE#6* and wild type (WT) leaves. Results were normalized against the expression of *EF1αA4* as an internal control. Values are means ± SD (n = 3). Asterisks indicate significant differences compared with the wild type (two-tailed paired Student's *t* test, $P \leq 0.05$).
